# Adverse drug reactions in geriatric psychiatry—retrospective cohort study of a 6-year period

**DOI:** 10.1007/s11845-023-03300-1

**Published:** 2023-02-20

**Authors:** Johannes Heck, Nina Noltemeyer, Martin Schulze Westhoff, Stephanie Deest-Gaubatz, Sebastian Schröder, Benjamin Krichevsky, Nicolas Simon, Swetlana Gerbel, Maximilian Friedrich, Dirk O. Stichtenoth, Stefan Bleich, Helge Frieling, Adrian Groh

**Affiliations:** 1 Institute for Clinical Pharmacology, Hannover Medical School, Carl-Neuberg-Str. 1, 30625 Hannover, Germany; 2 Department of Psychiatry, Social Psychiatry and Psychotherapy, Hannover Medical School, Hannover, Germany; 3 Institute for General Practice and Palliative Care, Hannover Medical School, Hannover, Germany; 4 Medical Service of the German Armed Forces, Kiel, Germany; 5 Hannover Medical School, MHH Information Technology, Hannover, Germany; 6 Department of Neurology, University Hospital Würzburg, Würzburg, Germany; 7 Drug Commissioner of Hannover Medical School, Hannover, Germany

**Keywords:** Adverse drug reactions, Drug safety, Elderly, Geriatric psychiatry, Potentially inappropriate prescribing

## Abstract

**Objective:**

To investigate the frequency and characteristics of adverse drug reactions (ADRs) that occurred on the gerontopsychiatric ward of Hannover Medical School over a 6-year period.

**Design:**

Retrospective monocentric cohort study.

**Results:**

Six hundred thirty-four patient cases (mean age 76.6 ± 7.1 years; 67.2% female) were analysed. In total, 92 ADRs in 56 patient cases were registered in the study population. The overall ADR prevalence, the ADR prevalence upon hospital admission, and the ADR prevalence during hospitalisation were 8.8%, 6.3%, and 4.9%, respectively. The most frequent ADRs were extrapyramidal symptoms, alterations in blood pressure or heart rate, and electrolyte disturbances. Of note, two cases of asystole and one case of obstructive airway symptoms related to general anaesthesia in the context of electroconvulsive therapy (ECT) were detected. The presence of coronary heart disease was associated with an increased risk of ADR occurrence (odds ratio (OR) 2.92, 95% confidence interval (CI) 1.37–6.22), while the presence of dementia was associated with a decreased risk of ADR development (OR 0.45, 95% CI 0.23–0.89).

**Conclusions:**

Type and prevalence of ADRs in the present study were largely in accordance with previous reports. By contrast, we did not observe a relationship between advanced age or female sex and ADR occurrence. We detected a risk signal for cardiopulmonary ADRs related to general anaesthesia in the context of ECT that warrants further investigation. Elderly psychiatric patients should be carefully screened for cardiopulmonary comorbidities before initiation of ECT.

**Supplementary Information:**

The online version contains supplementary material available at 10.1007/s11845-023-03300-1.

## Introduction

An adverse drug reaction (ADR) can be defined as “an appreciably harmful or unpleasant reaction, resulting from an intervention related to the use of a medicinal product, which predicts hazard from future administration and warrants prevention or specific treatment, or alteration of the dosage regimen, or withdrawal of the product” [1].

The European Commission estimates that approximately 5% of all ADRs lead to hospitalisation and that 5% of inpatients develop an ADR during their hospital stay. Moreover, ADRs constitute the fifth leading cause of death in hospital [2]. The risk of ADR occurrence has been reported to increase significantly with advancing age due to age-associated multimorbidity and polypharmacy [3, 4], inadequate prescribing [5], and insufficient monitoring of medications [6]. In addition, age-related physiological changes in pharmacokinetics and pharmacodynamics are considered to increase the likelihood of ADR occurrence in elderly people [7]. In the European Union, over 20% of citizens are 65 years of age or older, and in the next five decades, the proportion of this age group is expected to rise by one-third [8]. A study that investigated mental disorders in the German population found that 20% of people aged 65–79 years suffer from a mental illness [9, 10].

Extensive information about ADRs in the elderly general population is available. A meta-analysis by Oscanoa and colleagues concluded that nearly one in ten hospital admissions in older people was due to an ADR [11]. Alhawassi and co-workers estimated that 11.5% of patients experienced an ADR during hospitalisation [12]. In a recent systematic review and meta-analysis by Jennings et al., the prevalence of ADRs in hospitalised older adults was found to be even higher (16%), and five clinical presentations—fluid and electrolyte disturbances, gastrointestinal motility and defaecation disorders, renal disorders, hypotension/blood pressure dysregulation disorders/shock, and delirium—accounted for almost half of all ADRs [13].

By contrast, there is a paucity of data about the frequency and characteristics of ADRs in elderly patients suffering from psychiatric disorders. A large German registry study showed that people with severe mental illness suffer more frequently from somatic comorbidities compared to people without severe mental illness, leading to an elevated mortality rate and a loss of up to 12.3 life years [14]. Furthermore, the use of psychotropic drugs is associated with an increased risk of many somatic disorders [15].

Consequently, elderly people suffering from psychiatric disorders must be considered a vulnerable patient population that is particularly susceptible to the occurrence of ADRs. In order to protect elderly psychiatric patients more effectively from the occurrence of ADRs and their sequelae, it is of paramount importance to dissect the precise causes of ADRs in this population. The aim of the present study was to investigate the frequency and characteristics of ADRs in a cohort of elderly psychiatric inpatients over a 6-year period. Based on the study results, appropriate ADR prevention strategies shall be devised in the future.

## Methods

### Ethics approval

This study was approved by the Ethics Committee of Hannover Medical School (No. 9496_BO_K_2020) and was conducted in accordance with the Declaration of Helsinki (1964) and its later amendments (current version from 2013).

### Study design and study site

The study was designed as a retrospective monocentric cohort study of a 6-year period (01 January 2014 to 31 December 2019). The study was carried out at the gerontopsychiatric ward (27-bed facility) of the Department of Psychiatry, Social Psychiatry and Psychotherapy of Hannover Medical School (a university hospital in northern Germany).

### Eligibility criteria

Patients were eligible for enrolment in the study (a) if they were ≥ 65 years of age, (b) if they were treated on the gerontopsychiatric ward of the Department of Psychiatry, Social Psychiatry and Psychotherapy of Hannover Medical School between 01 January 2014 and 31 December 2019, and (c) if they or their legal representative had provided written informed consent for the use of patient-related data for research purposes.

### Patient selection and data acquisition

The study population was identified by the Enterprise Clinical Research Data Warehouse (ECRDW) [16] using structured medical data such as demographic characteristics, diagnoses, basic information on medications, and laboratory values. The ECRDW is a medical data repository which is maintained by Hannover Medical School (MHH) Information Technology and which comprises data from over 2.2 million patients. As one of the largest medical data repositories worldwide, the ECRDW has contributed to more than 300 clinical research projects of various medical disciplines (e.g. refs. [17–19]). Details on patient medication—such as dosages, mode of administration, and frequency of administration—were additionally extracted from unstructured medical reports by means of natural language processing [20].

### Identification, assessment, and validation of adverse drug reactions

Patient records (including hospital discharge letters) of all patients in the study population were manually screened for ADRs by the joint first authors (JH, NN), and identified ADRs were discussed with the senior author (AG), taking into account details on medication. All ADRs were subsequently validated by an interdisciplinary expert panel comprising specialists in internal medicine (BK), neurology (MF), clinical pharmacology (DOS), and psychiatry (MSW, SDG, SS, SB, HF).

### Drug interaction checks

Drug interaction checks were conducted with the electronic drug information system *Arzneimittel-Informations-Dienste* (AiD) *Klinik*^®^ (Dosing GmbH, Heidelberg, Germany).

### Assessment of potentially inappropriate medications for elderly patients

Potentially inappropriate medications for elderly patients (PIMs) were assessed with the aid of the PRISCUS list (*priscus* (Latin), ancient, venerable) [21] and the FORTA (Fit fOR The Aged) classification system (version 2021) [22, 23], both of which apply to people ≥ 65 years of age.

The PRISCUS list, which is specifically tailored to the German pharmaceutical market, tabulates a total of 83 PIMs and provides examples of suitable pharmacological alternatives for these PIMs [21]. Drugs used in the study population were categorised as “PRISCUS-listed” (i.e. PIMs), “Not PRISCUS-listed” (i.e. non-PIMs), or “No assignment possible”. The PRISCUS list classifies certain drugs (such as haloperidol, olanzapine, and zopiclone) as PIMs only if their cumulative daily dosages exceed specific thresholds (e.g. 2 mg, 10 mg, and 3.75 mg for haloperidol, olanzapine, and zopiclone, respectively). If the daily dosage of one of these drugs could not be retrieved from the patient records, the drug was categorised as “No assignment possible”.

The FORTA classification system (version 2021) [22, 23] was applied to assess the drugs used in the study population based on their main therapeutic indications. Drugs were categorised as A (i.e. indispensable drugs in the pharmacological treatment of elderly people), B (i.e. drugs with proven or obvious efficacy in elderly people), C (i.e. drugs with questionable efficacy–safety profiles in elderly people), or D (i.e. drugs that should be avoided in elderly people) [24]. If a drug was not listed in the FORTA classification system, it was classified as “Not labelled”.

### Statistical analyses

Quantitative variables are shown as means ± standard deviations or as medians with interquartile ranges (IQRs). For categorical variables, absolute and relative frequencies are reported. To investigate associations of sex and comorbidities with ADR occurrence, odds ratios (ORs) with corresponding 95% confidence intervals (CIs) were calculated. As the nature of our study was exploratory and hypothesis generating, no adjustments for multiple testing were carried out. Age distributions of patients with and without ADRs were tested for normality by utilisation of the Shapiro–Wilk test and by inspection of the histograms and Q–Q plots. Since the age distributions did not follow a normal distribution, median ages of patients with and without ADRs were compared with a two-sided Mann–Whitney *U* test. A *P* value < 0.05 was considered statistically significant. All statistical analyses were conducted with IBM^®^ SPSS^®^ Statistics for Windows, version 28 (Armonk, New York, USA).

## Results

### Study population

Overall, 634 patient cases involving 481 individual patients were identified by ECRDW and were manually screened for ADRs. The higher number of patient cases as compared to the number of individual patients is explained by returners. As ADRs may have occurred during every hospital stay of a returning patient, each case was evaluated separately. Therefore, the following statistical analyses refer to *n* = 634 as denominator unless stated otherwise. The mean age of the study population was 76.6 ± 7.1 years (median age 76 years, IQR 71–81 years, range 65–99 years), and more than two-thirds (67.2%; 426/634) of the patients were female. Overall, the study population was characterised by a high burden of both somatic and psychiatric diseases (Table 1). The most prevalent somatic and psychiatric diseases were arterial hypertension and depression, affecting 54.3% (344/634) and 39.1% (248/634) of the study population, respectively.

**Table 1:** Characteristics of the study population (*n* = 634)

**Variables**	***n***	%
**Sex**		
Female	426	67.2
Male	208	32.8
**Somatic diseases**^**a**^		
Arterial hypertension	344	54.3
Coronary heart disease	49	7.7
Chronic heart failure	55	8.7
Atrial fibrillation	100	15.8
Other cardiovascular disease (≥ 1 diagnosis)	247	39.0
Type-2 diabetes mellitus	101	15.9
Dyslipidaemia(s) (≥ 1 diagnosis)	60	9.5
Gastrointestinal, pancreatic, liver or gallbladder disease(s) (≥ 1 diagnosis)	92	14.5
Kidney disease(s) (≥ 1 diagnosis)	104	16.4
Disorder(s) of the genitourinary system (≥ 1 diagnosis)	99	15.6
Pulmonary disease(s) (≥ 1 diagnosis)	77	12.1
Neurological disorder(s) (≥ 1 diagnosis)	267	42.1
Otorhinolaryngologic disease(s) (≥ 1 diagnosis)	51	8.0
Ophthalmic disease(s) (≥ 1 diagnosis)	58	9.1
Dermatologic disease(s) (≥ 1 diagnosis)	62	9.8
Thyroid disease(s) (≥ 1 diagnosis)	109	17.2
Infectious disease(s) (≥ 1 diagnosis)	93	14.7
Disturbance(s) of fluid and/or electrolyte balance(s) (≥ 1 diagnosis)	122	19.2
Vitamin deficiency/deficiencies (≥ 1 diagnosis)	47	7.4
Trauma(ta) (≥ 1 diagnosis)	62	9.8
Other somatic diagnosis/diagnoses	232	36.6
**Psychiatric diseases**^**a**^		
Depression^b^	248	39.1
Bipolar affective disorder^c^	75	11.8
Schizophrenia or schizophreniform disorder^d^	126	19.9
Dementia^e^	215	33.9
Delirium^f^	111	17.5
Mental and behavioural disorder due to use of alcohol, tobacco, sedatives and hypnotics, or opioids^g^ (≥ 1 diagnosis)	126	19.9
Other psychiatric diagnosis/diagnoses	157	24.8

^a^Patients could have more than one diagnosis

^b^ICD-10 F32, F33

^c^ICD-10 F31

^d^ICD-10 F06.2, F2X

^e^ICD-10 F00, F01, F02, F03

^f^ICD-10 F05

^g^ICD-10 F10, F11, F13, F17. ICD-10 denotes International Statistical Classification of Diseases and Related Health Problems 10th Revision

### Adverse drug reactions

In total, 92 ADRs were registered in 56 of 634 patient cases, yielding an overall ADR prevalence of 8.8% (56/634). In 29 of the 56 patient cases affected by ADRs (51.8%), one ADR was detected; in 19 cases (33.9%), two ADRs were identified; in seven cases (12.5%), three ADRs were registered; and in one case (1.8%), four ADRs were observed.

Remarkably, in 6.3% (40/634) of patient cases, ADRs were identified immediately upon hospital admission. Of note, in nearly half of patient cases with ADRs detected upon hospital admission (42.5%; 17/40), the detected ADRs were responsible for or contributed to patients’ hospitalisations. The prevalence of ADRs that occurred in hospital was 4.9% (31/634).

Extrapyramidal symptoms represented the most frequent type of ADRs in the study population (20.7%; 19/92), comprising Parkinsonism (*n* = 9), extrapyramidal side effects, not otherwise specified (*n* = 6), and tardive dyskinesia/dystonia (*n* = 4) (Table 2). Alterations in blood pressure or heart rate constituted the second most frequent group of ADRs (15.2%; 14/92); within this group, (orthostatic) hypotension (*n* = 6) and circulatory disturbances, not otherwise specified (*n* = 5) dominated, while tachycardia, hypertension, and QT_c_ interval prolongation were less frequently observed (one case each). Electrolyte disturbances accounted for the third largest group of ADRs (10.9%; 10/92), and comprised syndrome of inappropriate antidiuretic hormone secretion (SIADH; *n* = 4), hyponatraemia (*n* = 2), hypokalaemia, hyperkalaemia, oedema, and diabetes insipidus (one case each).

**Table 2:** Absolute and relative frequencies of adverse drug reactions (*n* = 92) detected in the study population

**Adverse drug reactions**	***n***	%^a^
Extrapyramidal symptoms	19	20.7
Alterations in blood pressure or heart rate	14	15.2
Electrolyte disturbances	10	10.9
Hypersensitivity reactions	6	6.5
Delirium	5	5.4
Disturbances of salivation	5	5.4
Somnolence	4	4.3
Impairment of renal function and urinary tract-related symptoms	3	3.3
Agitation	3	3.3
Asystole in the context of electroconvulsive therapy	2	2.2
Constipation	2	2.2
Exacerbation of psychotic symptoms	2	2.2
Hallucinations	2	2.2
Blood dyscrasias	2	2.2
Respiratory symptoms^b^	2	2.2
Intoxication (accidental)	2	2.2
Polyneuropathy	2	2.2
Depression	1	1.1
Disturbance of central thermoregulation	1	1.1
Cognitive dysfunction	1	1.1
Hypothyroidism	1	1.1
Hyperhidrosis	1	1.1
Exacerbation of dysarthrophonia	1	1.1
Other	1	1.1

^a^Percentages may not total 100 due to rounding

^b^One respiratory symptom occurred in the context of electroconvulsive therapy

### Associations of sex, age, and comorbidities with ADR occurrence

Female sex, advanced age, and certain comorbidities have been reported as risk factors for ADR occurrence [12]. To investigate possible associations of the cited parameters with ADR occurrence in the present study, corresponding ORs were calculated. Female sex was not associated with an increased risk of ADR occurrence in the present study (OR 1.22, 95% CI 0.67–2.24). Similarly, no statistically significant difference was observed between the median age of patients with ADRs and the median age of patients without ADRs (77 years (IQR 70.5–83.75 years) vs. 76 years (IQR 71–81 years), *P* = 0.24). The presence of coronary heart disease was associated with an increased risk of ADR occurrence (OR 2.92, 95% CI 1.37–6.22), whereas no increased risk was observed for arterial hypertension (OR 1.44, 95% CI 0.82–2.54), chronic heart failure (OR 1.58, 95% CI 0.68–3.67), atrial fibrillation (OR 1.91, 95% CI 1.00–3.64), type-2 diabetes mellitus (OR 1.01, 95% CI 0.48–2.14), depression (OR 1.62, 95% CI 0.93–2.80), bipolar affective disorder (OR 0.89, 95% CI 0.37–2.14), or schizophrenia (OR 1.11, 95% CI 0.57–2.17). Astonishingly, the presence of dementia was associated with a reduced risk of ADR occurrence (OR 0.45, 95% CI 0.23–0.89).

### Suspected drugs

**Fig. 1:**
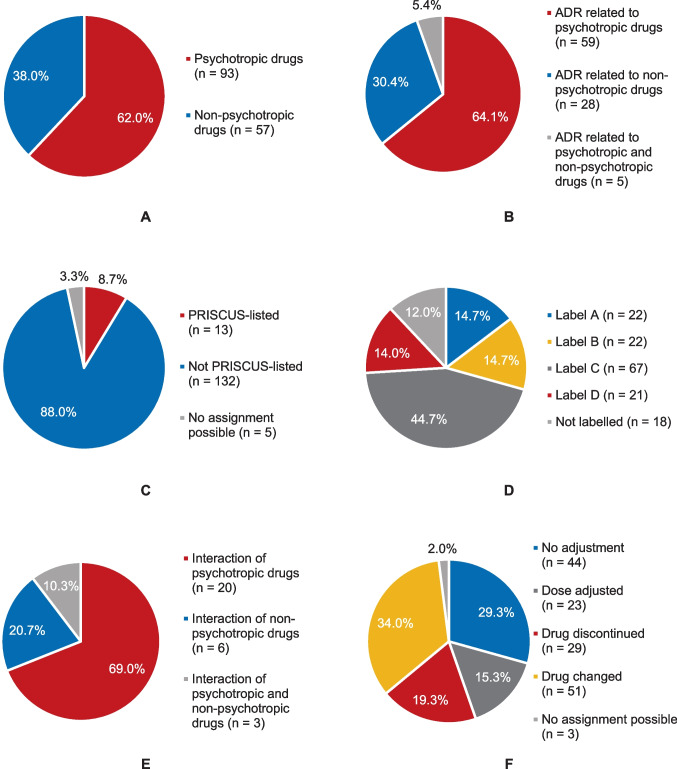
Analysis of drugs with a suspected relationship to adverse drug reactions in the study population. **A** Differentiation of drugs (*n* = 150) with a suspected relationship to ADRs into psychotropic and non-psychotropic substances. **B** Relatedness of ADRs (*n* = 92) to psychotropic and non-psychotropic drugs. **C** PRISCUS listing of drugs (*n* = 150) with a suspected relationship to ADRs (“PRISCUS-listed” refers to drugs that are considered potentially inappropriate medications for elderly patients according to the PRISCUS list). **D** FORTA labelling of drugs (*n* = 150) with a suspected relationship to ADRs (Label A: indispensable drugs in the pharmacological treatment of elderly people; Label B: drugs with proven or obvious efficacy in elderly people; Label C: drugs with questionable efficacy–safety profiles in elderly people; Label D: drugs that should be avoided in elderly people; “Not labelled”: drug not mentioned in the FORTA classification system). **E** Characterisation of drug–drug interactions that were likely related to the occurrence of 29 ADRs. **F** Adjustment of drugs (*n* = 150) with a suspected relationship to ADRs after the ADR had occurred. ADR denotes adverse drug reaction, FORTA Fit fOR The Aged

Overall, 150 drugs with a suspected relationship to the detected ADRs were identified, 62.0% (93/150) of which were characterised as psychotropic drugs (Fig. 1A). Psychotropic drugs most frequently implicated in ADR occurrence were second-generation antipsychotics (total, *n* = 36; more specifically risperidone, *n* = 11; olanzapine, *n* = 7; quetiapine, *n* = 7; aripiprazole, *n* = 6; clozapine, *n* = 3; and amisulpride, *n* = 2), followed by antidepressants (total, *n* = 28; more specifically selective serotonin–norepinephrine reuptake inhibitors, *n* = 9; tricyclic antidepressants, *n* = 8; selective serotonin reuptake inhibitors, *n* = 6; and tetracyclic antidepressants, *n* = 5), and first-generation antipsychotics (total, *n* = 18; more specifically pipamperone, *n* = 8; haloperidol, *n* = 4; promethazine, *n* = 3; melperone, *n* = 2; and loxapine, *n* = 1) (Table 3 and Supplementary Table 1). Among non-psychotropic drugs, antihypertensive agents (*n* = 30) were primarily suspected to be responsible for ADR occurrence, more specifically diuretics (*n* = 14), angiotensin-converting enzyme inhibitors (*n* = 8), beta blockers (*n* = 5), and angiotensin II receptor blockers (*n* = 3).

**Table 3:** Absolute and relative frequencies of drugs (*n* = 150) with a suspected relationship to adverse drug reactions. For a listing of the individual substances please refer to Supplementary Table 1

**Drugs with a suspected relationship to ADR occurrence**	***n***	%^a^
Second-generation antipsychotics	36	24.0
Antihypertensives	30	20.0
Antidepressants	28	18.7
First-generation antipsychotics	18	12.0
Anticonvulsants	6	4.0
Anti-infectives for systemic use	5	3.3
Opioids	5	3.3
Lithium	4	2.7
Digitalis glycosides	3	2.0
Anaesthetics	2	1.3
Antiparkinson drugs	2	1.3
Chemotherapeutics	2	1.3
Dalteparin	1	0.7
Ketamine	1	0.7
Levothyroxine	1	0.7
Lorazepam	1	0.7
Prednisolone	1	0.7
Succinylcholine	1	0.7
Tamsulosin	1	0.7
Thiamazole	1	0.7
Zopiclone	1	0.7

*ADR* denotes adverse drug reaction

^a^Percentages may not total 100 due to rounding

Fifty-nine of 92 ADRs (64.1%) were presumably elicited exclusively by psychotropic drugs, while 28 ADRs (30.4%) were presumably evoked exclusively by non-psychotropic drugs. Five ADRs (5.4%) were suspected to be caused by a combination of psychotropic and non-psychotropic drugs (Fig. 1B).

Of the 150 drugs with a suspected relationship to ADR occurrence, 23 drugs (15.3%) were adjusted in dose after the ADR had occurred, 29 drugs (19.3%) were discontinued, 51 drugs (34.0%) were switched to another substance, and 44 drugs (29.3%) were left unchanged (Fig. 1F).

### Drug–drug interactions

To investigate a possible link between drug–drug interactions (DDIs) and ADR occurrence, the medication of patients affected by (an) ADR(s) were scrutinised for potential DDIs by utilisation of AiD*Klinik*^®^. DDIs were likely involved in the occurrence of 29 of 92 ADRs (31.5%). In 20 of these 29 ADRs (69.0%), DDIs were elicited exclusively by combinations of psychotropic drugs (Fig. 1E).

### Potentially inappropriate medications for elderly patients

PIM prescriptions have been recognised as a significant contributor to ADR development in elderly people [25–27]. To investigate the role of PIMs in ADR development in the present study, drugs (*n* = 150) with a suspected relationship to ADRs were assessed for PIM prescriptions with the aid of the PRISCUS list and the FORTA classification system. Thirteen of the 150 suspected agents (8.7%) were designated as PIMs according to the PRISCUS list: amitriptyline (*n* = 3), clozapine (*n* = 3), digoxin (*n* = 2), doxepin (*n* = 1), haloperidol (at a daily dose of more than 2 mg; *n* = 1), olanzapine (at a daily dose of more than 10 mg; *n* = 1), trimipramine (*n* = 1), and zopiclone (at a daily dose of more than 3.75 mg; *n* = 1).

Sixty-seven (44.7%) and 21 (14.0%) of the 150 suspected agents were designated as category C drugs and category D drugs according to the FORTA classification system, respectively (Fig. 1D). The most frequent category C and category D drugs with a suspected relationship to ADRs were risperidone (*n* = 11) and aripiprazole (*n* = 6), respectively (Table 4).

**Table 4:** Absolute and relative frequencies of FORTA category C drugs (i.e. drugs with questionable efficacy–safety profiles in elderly people) and FORTA category D drugs (i.e. drugs that should be avoided in elderly people) with a suspected relationship to adverse drug reactions in the study population

**FORTA category C and D drugs with a suspected relationship to ADR occurrence**	***n***	%^a^
**FORTA category C drugs**	**67**	**100**
Risperidone	11	16.4
Pipamperone	8	11.9
Olanzapine	7	10.4
Quetiapine	6	9.0
Venlafaxine	6	9.0
Mirtazapine	5	7.5
Spironolactone	4	6.0
Amitriptyline	3	4.5
Duloxetine	3	4.5
Digoxin	2	3.0
Melperone	2	3.0
Tramadol	2	3.0
Valproic acid	2	3.0
Digitoxin	1	1.5
Doxepin	1	1.5
Gabapentin	1	1.5
Oxycodone	1	1.5
Tilidine	1	1.5
Zopiclone	1	1.5
**FORTA category D drugs**	**21**	**100**
Aripiprazole	6	28.6
Haloperidol	4	19.0
Carbamazepine	3	14.3
Clozapine	3	14.3
Opipramol	3	14.3
Ciprofloxacin	1	4.8
Prednisolone	1	4.8

*ADR* denotes adverse drug reaction, *FORTA* Fit fOR The Aged

^a^Percentages may not total 100 due to rounding

## Discussion

While ADRs have been studied extensively in the older general population [28], data about the frequency and characteristics of ADRs in elderly patients suffering from mental illness are limited. The present study aimed at diminishing this knowledge gap by providing a detailed analysis of ADRs that occurred over a 6-year period in a population of elderly psychiatric inpatients treated on the gerontopsychiatric ward of a university hospital in northern Germany. We detected an overall ADR prevalence of 8.8%. The ADR prevalence upon hospital admission and the ADR prevalence during hospitalisation were calculated as 6.3% and 4.9%, respectively.

Whereas the ADR prevalence upon hospital admission in the present study (6.3%) was largely in accordance with figures put forward by Bouvy and colleagues (median 3.6%, mean 4.6%) [29], the in-hospital ADR prevalence in our study (4.9%) was markedly lower than in systematic reviews and meta-analyses by Alhawassi et al. (11.5%) [12] and Jennings et al. (16%) [13]. However, it must be considered that there was a substantial degree of heterogeneity between the studies included in these systematic reviews/meta-analyses, leading to wide 95% CIs (0–27.7% in the Alhawassi et al. systematic review [12]; 12–22% in the Jennings et al. meta-analysis [13]). In addition, the systematic reviews and meta-analyses by Alhawassi et al. [12] and Jennings et al. [13] included data from studies across different medical disciplines, yielding a much broader spectrum of ADRs as compared to our study, which only enrolled elderly psychiatric inpatients.

Another explanation for the comparatively lower in-hospital ADR prevalence in our study might be that older psychiatric patients are more acquainted with the (side) effects of psychotropic drugs and that they do not communicate clinical symptoms as readily to their treating physicians as their younger counterparts [30]. Moreover, Greil and colleagues suggested that physicians may avoid medications with a history of intolerance in older patients, while younger patients may receive certain drugs for the first time and may therefore develop ADRs more frequently compared to older patients [30]. Besides, older psychiatric patients often display a variety of somatic complaints, making it more difficult to establish a link between a reported symptom and a specific medication [30]. In summary, these factors may have led to the comparatively low in-hospital ADR prevalence in the present study.

Extrapyramidal symptoms, alterations in blood pressure or heart rate, and electrolyte disturbances represented the three most frequent types of ADRs in our study. Taken together, these three ADR groups accounted for approximately half (46.7%) of all ADRs registered in the study population. These ADRs represent well-known side effects of psychotropic medications. Antipsychotics, especially first-generation agents, are notorious for causing extrapyramidal symptoms [31–33]. Moreover, antipsychotics—but also tricyclic antidepressants—are known to elicit alterations in blood pressure and/or heart rate [34], especially if co-prescribed with other drugs acting on the cardiovascular system, e.g. antihypertensive agents [35]. Selective serotonin reuptake inhibitors and selective serotonin–norepinephrine reuptake inhibitors may evoke electrolyte disturbances such as hyponatraemia, especially when co-administered with thiazide diuretics [35].

Even though the majority (62.0%) of the drugs that were likely related to ADR occurrence in our study were classified as psychopharmaceuticals, a considerable share (i.e. 38.0%) were non-psychotropic drugs. This demonstrates that medications for the treatment of somatic diseases must always be taken into consideration alongside psychotropic drugs when the risk of ADRs is evaluated in elderly psychiatric patients.

Drug–drug interactions were implicated in the development of 31.5% of the ADRs in our study population, most notably interactions between psychotropic drugs. This finding is in accordance with previous reports that suggested DDIs as a risk factor for ADR occurrence [12] and that described a high DDI potential of psychotropic drugs [35].

Astonishingly, we observed marked discrepancies between the PRISCUS list and the FORTA classification system when evaluating the 150 drugs that were likely related to ADR occurrence in terms of their general appropriateness for elderly patients. Whereas 8.7% of the drugs with a suspected relationship to ADR occurrence were designated as PIMs according to the PRISCUS list [21], 58.7% were classified as unsuitable for elderly people according to the FORTA classification system (i.e. FORTA categories C plus D) [22, 23]. This finding suggests that the FORTA classification system has a higher sensitivity in the detection of potentially inappropriate prescribing for elderly psychiatric inpatients compared to the PRISCUS list. This divergence may be explained by the fact that the PRISCUS list has remained unaltered since its inception in 2010, whereas the FORTA classification system has been updated at regular intervals, its latest version dating from 2021. Tools for medication reviews, especially tools for the assessment of potentially inappropriate prescribing, need to be kept up to date in order to allow for reliable therapeutic conclusions to be drawn. It must be annotated that the PRISCUS list is currently being revised. A release date of the PRISCUS list 2.0, however, has not yet been officially announced [36].

Only one in five drugs likely related to ADR occurrence was discontinued after the ADR had occurred, as opposed to nearly 30% of the drugs with a suspected relationship to ADRs which were left unchanged after the event. This demonstrates that in geriatric psychiatry therapeutic alternatives are often sparse or even lacking altogether. Consequently, ADRs of limited clinical severity must sometimes be tolerated by both patient and treating physician(s) in order to safeguard a sufficiently effective pharmacotherapy. To abstain from pharmacotherapy often is not a feasible option in geriatric psychiatry. Fortunately, in the present study, more than every third drug with a suspected relationship to ADR occurrence could be replaced with an adequate pharmacological alternative.

Female sex has repeatedly been reported as a risk factor for ADR occurrence [12], whereas in the present study, we did not observe such an association. By contrast, the presence of coronary heart disease was associated with an increased risk of ADR occurrence. This finding is in accordance with previous reports which described cardiovascular disease as a risk factor for ADRs [12]. However, other cardiovascular diseases such as arterial hypertension, chronic heart failure, and atrial fibrillation were not associated with an increased risk of ADR occurrence in the present study. In contrast to previous accounts, which cited dementia as a risk factor for ADR development [12], the presence of dementia was associated with a reduced risk of ADR occurrence in our study. We hypothesise that the reduced ability of patients suffering from dementia to verbalise and to communicate physical complaints to their treating physicians may have led to an underreporting of ADRs as compared to non-demented patients. However, it must be underscored that no adjustments for multiple testing were conducted for the ORs in our study (see also the paragraph “Statistical analyses” in the “Methods” section); therefore, these findings must be interpreted with circumspection and should be validated in a future prospective study.

Of particular clinical importance, the majority of patient cases with ADRs were not identified during inpatient treatment but were already present upon admission to the hospital (6.3%; 40/634). Of these, nearly half (42.5%; 17/40) were responsible for patients’ hospital admissions. We speculate that the high ADR detection rate immediately upon hospital admission may be explained by the high priority of pharmacotherapy safety and pharmacovigilance at our institution. Hannover Medical School was the first German university hospital to establish a professorship for drug safety in 2011 [37]. The pharmacological expertise at Hannover Medical School is integrated in and coordinated by the Centre for Drug Safety (*Zentrum für Arzneimittelsicherheit*, ZAS), an interdisciplinary and multiprofessional platform which comprises specialists from clinical pharmacology, microbiology, hospital epidemiology, transfusion medicine, pharmacy, clinical chemistry, psychiatry, and general practice, as well as the office of the Drug Commissioner [38]. Through ZAS, regular training programmes such as lectures, seminars, and clinical rotations/internships as well as continuing medical education activities are offered to both physicians and medical students. Furthermore, the trinational (Germany, Austria, and Switzerland) pharmacovigilance programme Drug Safety in Psychiatry (*Arzneimittelsicherheit in der Psychiatrie*, AMSP), which aims at improving drug safety in psychiatric inpatients, has its headquarters at and coordinates its activities from Hannover Medical School [39]. Besides other activities, AMSP organises regular case conferences on a regional and supraregional level, where (suspected) ADR cases are being discussed by experts in the field.

Electroconvulsive therapy (ECT) is generally considered an effective and well-tolerated therapeutic modality in the treatment of older patients with major depression and other psychiatric disorders [34, 40, 41]. However, we registered three ADRs (two asystoles and one case of obstructive airway symptoms) related to general anaesthesia in the context of ECT. This risk signal warrants further investigation. We suggest a diligent benefit–risk evaluation and a thorough screening for cardiopulmonary comorbidities (e.g. by electrocardiography and chest X-ray) in elderly psychiatric patients prior to the initiation of ECT.

Limitations of the present study mainly arise from its monocentric and retrospective design. However, due to the long observation period (i.e. 6 years) and the size of the study population (*n* = 634), we think that our study results are overall representative of elderly psychiatric patients treated in German university hospitals. The ADRs analysed in this study were retrieved from patient records, in particular from hospital discharge letters, an approach that has been utilised in similar form in previous studies [42, 43]. It may be assumed that only ADRs with a certain severity were deemed as sufficiently clinically relevant by the treating physician(s) to be mentioned and described in further detail in the hospital discharge letter and hence to be communicated to the patient’s general practitioner or ambulatory psychiatrist/neurologist. A certain degree of underreporting of ADRs, especially ADRs of mild severity, must therefore be assumed in this study. Notwithstanding, the ADR documentation via hospital discharge letters adequately reflects treating physicians’ approach to, assessment and documentation of ADRs during clinical routine.

The primary objective of our study, i.e. the estimation of the frequency and characterisation of ADRs that occurred on the gerontopsychiatric ward of our institution over the course of 6 years, was achieved. However, our study was non-interventional. The effectiveness of interventions to optimise pharmacotherapy of elderly people in order to reduce ADRs was shown by Gray and colleagues in a meta-analysis of 13 randomised controlled trials. Participants in the intervention group were 21% less likely to experience an ADR compared to those in the control group (OR = 0.79; 95% CI = 0.62–0.99). The benefit was even more pronounced for the reduction of serious ADRs (OR = 0.64, 95% CI = 0.42–0.98) [44]. A similar intervention—i.e. an interdisciplinary ward round with medication review conducted by a team of specialists in psychiatry, internal medicine, geriatrics, neurology, and clinical pharmacology—has already been tested at our institution in a pilot study [45]. This pilot study, however, did not include a control group. Hence, in order to investigate if the in-hospital ADR rate can be reduced by this intervention, a randomised controlled study is currently being devised at our institution.

### Supplementary Information

Below is the link to the electronic supplementary material.

Supplementary file1 (DOCX 42 KB)

## Data Availability

The data that support the findings of this study are available upon reasonable request from the corresponding author.

## Abbreviations

ADR: Adverse drug reaction
AiD: *Arzneimittel-Informations-Dienste* (Drug Information Services)
AMSP: *Arzneimittelsicherheit in der Psychiatrie* (Drug Safety in Psychiatry)
CI: Confidence interval
DDI: Drug–drug interaction
ECRDW: Enterprise Clinical Research Data Warehouse
ECT: Electroconvulsive therapy
FORTA: Fit fOR The Aged
ICD-10: International Statistical Classification of Diseases and Related Health Problems 10th Revision
IQR: Interquartile range
OR: Odds ratio
PIM: Potentially inappropriate medication (for elderly patients)
ZAS: *Zentrum für Arzneimittelsicherheit* (Centre for Drug Safety)
