# Supplementary material for: Adverse drug reactions in geriatric psychiatry—retrospective cohort study of a 6-year period
Source: Ir J Med Sci. 2023 Feb 20;192(6):2917–27. doi: 10.1007/s11845-023-03300-1 (PMC10692025; doi:10.1007/s11845-023-03300-1)
Supplement: Supplementary file 1 — Supplementary file1 (DOCX 42 KB) [file 11845_2023_3300_MOESM1_ESM.docx]

**SUPPLEMENTARY TABLE 1** Absolute and relative frequencies of drugs (n = 150) with a suspected relationship to adverse drug reactions

| **Drugs with a suspected relationship to ADR occurrence** | **n** | **%^a^** |
| --- | --- | --- |
| Risperidone | 11 | 7.3 |
| Pipamperone | 8 | 5.3 |
| Olanzapine | 7 | 4.7 |
| Quetiapine | 7 | 4.7 |
| Aripiprazole | 6 | 4.0 |
| Venlafaxine | 6 | 4.0 |
| Mirtazapine | 5 | 3.3 |
| Ramipril | 5 | 3.3 |
| Torasemide | 5 | 3.3 |
| Haloperidol | 4 | 2.7 |
| Lithium | 4 | 2.7 |
| Spironolactone | 4 | 2.7 |
| Amitriptyline | 3 | 2.0 |
| Carbamazepine | 3 | 2.0 |
| Clozapine | 3 | 2.0 |
| Duloxetine | 3 | 2.0 |
| Metoprolol | 3 | 2.0 |
| Opipramol | 3 | 2.0 |
| Promethazine | 3 | 2.0 |
| Sertraline | 3 | 2.0 |
| Amisulpride | 2 | 1.3 |
| Anaesthetics (not otherwise specified) | 2 | 1.3 |
| Bisoprolol | 2 | 1.3 |
| Chemotherapeutics (not otherwise specified) | 2 | 1.3 |
| Digoxin | 2 | 1.3 |
| Enalapril | 2 | 1.3 |
| Escitalopram | 2 | 1.3 |
| Furosemide | 2 | 1.3 |
| Melperone | 2 | 1.3 |
| Tramadol | 2 | 1.3 |
| Valproic acid | 2 | 1.3 |
| Acyclovir | 1 | 0.7 |
| Amlodipine | 1 | 0.7 |
| Benserazide | 1 | 0.7 |
| Buprenorphine | 1 | 0.7 |
| Candesartan | 1 | 0.7 |
| Cefuroxime | 1 | 0.7 |
| Ciprofloxacin | 1 | 0.7 |
| Citalopram | 1 | 0.7 |
| Dalteparin | 1 | 0.7 |
| Digitoxin | 1 | 0.7 |
| Doxepin | 1 | 0.7 |
| Gabapentin | 1 | 0.7 |
| Hydrochlorothiazide | 1 | 0.7 |
| Indapamide | 1 | 0.7 |
| Ketamine | 1 | 0.7 |
| Levodopa | 1 | 0.7 |
| Levothyroxine | 1 | 0.7 |
| Lorazepam | 1 | 0.7 |
| Loxapine | 1 | 0.7 |
| Olmesartan | 1 | 0.7 |
| Oxycodone | 1 | 0.7 |
| Perindopril | 1 | 0.7 |
| Piperacillin | 1 | 0.7 |
| Prednisolone | 1 | 0.7 |
| Succinylcholine | 1 | 0.7 |
| Tamsulosin | 1 | 0.7 |
| Tazobactam | 1 | 0.7 |
| Thiamazole | 1 | 0.7 |
| Tilidine | 1 | 0.7 |
| Trimipramine | 1 | 0.7 |
| Valsartan | 1 | 0.7 |
| Zopiclone | 1 | 0.7 |

^a^Percentages may not total 100 due to rounding.

ADR denotes adverse drug reaction.
